# Communication Bridge™-2 (CB2): an NIH Stage 2 randomized control trial of a speech-language intervention for communication impairments in individuals with mild to moderate primary progressive aphasia

**DOI:** 10.1186/s13063-022-06162-7

**Published:** 2022-06-13

**Authors:** Angela C. Roberts, Alfred W. Rademaker, Elizabeth Ann Salley, Aimee Mooney, Darby Morhardt, Melanie Fried-Oken, Sandra Weintraub, Marsel Mesulam, Emily Rogalski

**Affiliations:** 1 Pepper Department of Communication Sciences and Disorders, Northwestern University, Evanston, USA; 2 University of Western Ontario, School of Communication Sciences and Disorders and Department of Computer Science, Ontario, Canada; 3 Mesulam Center for Cognitive Neurology and Alzheimer’s Disease, Northwestern University, Chicago, USA; 4 Oregon Health & Science University, Portland, USA; 5 Mesulam Center for Alzheimer’s Disease and Cognitive Neurology and Department of Psychiatry and Behavioral Sciences, Northwestern University, Chicago, USA

**Keywords:** Primary progressive aphasia, Dementia, Telehealth, Speech, Language, Communication, Speech-language pathology, Randomized clinical trial, Aphasia, Therapy, Frontotemporal dementia, Behavioral intervention, Dyadic intervention, Alzheimer’s

## Abstract

**Background:**

Primary progressive aphasia (PPA) is a clinical dementia syndrome. Impairments in language (speaking, reading, writing, and understanding) are the primary and persistent symptoms. These impairments progress insidiously and devastate communication confidence, participation, and quality of life for persons living with PPA. Currently, there are no effective disease modifying treatments for PPA. Speech-language interventions hold promise for mitigating communication challenges and language symptoms. However, evidence regarding their efficacy in PPA is of low quality and there are currently no rigorous randomized trials.

**Method:**

Communication Bridge™-2 (CB2) is a Stage 2, superiority, single-blind, randomized, parallel group, active-control, behavioral clinical trial delivered virtually within a telehealth service delivery model to individuals with PPA. Ninety carefully characterized participants with clinically confirmed PPA will be randomized to one of two speech-language intervention arms: (1) Communication Bridge™ a dyadic intervention based in communication participation therapy models that incorporates salient training stimuli or (2) the control intervention a non-dyadic intervention based in impairment therapy models addressing word retrieval and language production that incorporates fixed stimuli. The superiority of Communication Bridge™ over the Control arm will be evaluated using primary outcomes of communication confidence and participation. Other outcomes include accuracy for trained words and scripts. Participants complete two therapy blocks over a 12-month period. Outcomes will be measured at baseline, at each therapy block, and at 12 months post enrollment.

**Discussion:**

The CB2 trial will supply Level 2 evidence regarding the efficacy of the Communication Bridge™ intervention delivered in a telehealth service delivery model for individuals with mild to moderate PPA. An important by-product of the CB2 trial is that these data can be used to evaluate the efficacy of speech-language interventions delivered in both trial arms for persons with PPA. The impact of these data should not be overlooked as they will yield important insights examining why interventions work and for whom, which will advance effectiveness trials for speech-language interventions in PPA.

**Trial Registration:**

ClinicalTrials.govNCT03371706. Registered prospectively on December 13, 2017.

## Administrative information

Note: the numbers in curly brackets in this protocol refer to SPIRIT checklist item numbers. The order of the items has been modified to group similar items (see http://www.equator-network.org/reporting-guidelines/spirit-2013-statement-defining-standard-protocol-items-for-clinical-trials/).

**Table:** 

Title {1}	Communication Bridge™-2 (CB2): An NIH Stage 2 randomized control trial of a speech-language intervention for communication impairments in individuals with mild to moderate primary progressive aphasia
Trial registration {2a and 2b}.	The CB2 trial is registered with ClinicalTrials.gov (NCT03371706).
Protocol version {3}	(Version 12.0 March 27, 2020)
Funding {4}	The clinical trial is funded by the National Institute on Aging (R01AG055425).
Author details {5a}	Angela C. Roberts, Northwestern University, Pepper Department of Communication Sciences and Disorders; University of Western Ontario, School of Communication Sciences and Disorders and Department of Computer Science Alfred W. Rademaker, PhD, Northwestern University, Mesulam Center for Cognitive Neurology and Alzheimer’s Disease Elizabeth Ann Salley, MA, Northwestern University, Mesulam Center for Cognitive Neurology and Alzheimer’s Disease Aimee Mooney, MSc-SLP, CCC-SLP, Oregon Health & Science University Darby Morhardt, PhD, LCSW, Northwestern University, Mesulam Center for Cognitive Neurology and Alzheimer’s Disease Melanie Fried-Oken, MSc-SLP, PhD, CCC-SLP, Oregon Health & Science University Sandra Weintraub, PhD, Northwestern University, Mesulam Center for Alzheimer’s Disease and Cognitive Neurology and Department of Psychiatry and Behavioral Sciences Marsel Mesulam, MD, Northwestern University, Mesulam Center for Cognitive Neurology and Alzheimer’s Disease and Department of Neurology Emily Rogalski, PhD, Northwestern University, Mesulam Center for Cognitive Neurology and Alzheimer’s Disease and Department of Psychiatry and Behavioral Sciences
Name and contact information for the trial sponsor {5b}	Northwestern University, Office of Sponsored Research 750 N. Lake Shore Drive Rubloff 7^th^ Floor Chicago, Illinois 60611 312-503-7955
Role of sponsor {5c}	Neither the study sponsor nor funder have any role in the study design, data collection, management analysis or interpretation of data. They were not involved in the writing of this report or the decision to submit the report for publication.

## Introduction

### Background and rationale {6a}

Communication Bridge™-2 (CB2) is an NIA (National Institute on Aging) funded Stage 2 [1], single-blind, randomized, parallel group, active-control, behavioral clinical trial delivered virtually within a telehealth service delivery model to individuals with primary progressive aphasia (PPA), a clinical neurodegenerative dementia syndrome marked by language decline. The CB2 trial is designed to test the central hypothesis that the Communication Bridge™ intervention is superior to the Control arm intervention for improving: (1) participation in everyday communication activities measured by the Communication Participation Item Bank (CPIB) [2] and communication participation goals using Goal Attainment Scaling (GAS) [3, 4] and (2) self-reported communication confidence measured by The Communication Confidence Rating Scale for Aphasia (CCRSA) [5, 6]. Ninety participants with mild-moderate primary PPA and their co-enrolled communication partners are randomly allocated to one of two active intervention arms. In the *experimental arm* (sample target = 54), participants receive Communication Bridge™, a multi-component, participation-focused [7], dyadic intervention in which both the person with PPA and their co-enrolled communication partner are intervention recipients. Communication Bridge™ is modeled on the Living with Aphasia: Framework for Outcome Measurement (A-FROM) [8–10] and the Care Pathway Model [11, 12]. Consistent with participation-focused intervention models [7], personally salient training stimuli are incorporated into all therapy activities in the Experimental arm. By contrast, the *control arm* (sample target = 36) includes a non-dyadic intervention in which the person with PPA is the active intervention recipient and their communication partner is in a supporting role. In the control arm, participants receive a speech-language intervention designed to address impairment and functional limitations, comprised of activities that address word retrieval and ‘automatic’ speech production using fixed, non-personalized, stimuli across participants. In the current trial, both arms are delivered via telehealth and are complemented by a self-paced home exercise program delivered online through the Communication Bridge™ web application. Dosing of synchronous speech-language intervention activities and the trial outcome measures are equivalent across trial arms. The CB2 trial will provide Level 2 evidence [13] regarding the efficacy of the Communication Bridge™ intervention delivered in a telehealth service delivery model for individuals with mild to moderate PPA.

#### Overview of primary progressive aphasia

PPA is a clinical dementia syndrome of neurodegenerative etiology [14]. Salient clinical symptoms of PPA include declines in understanding and expressing language through symbolic representations (e.g., words, sounds, letters) [15]. Language impairments remain the persistent and salient feature of PPA over the disease course [16–19]. With time, other cognitive and behavioral deficits can appear as disease spreads throughout the brain beyond the language network [19]. An estimated 60% of PPA is associated with a form of frontotemporal lobar degeneration and the remaining 40% with the neuropathology of Alzheimer’s disease [19, 20]. There are currently no effective disease modifying pharmacologic or behavioral treatments. PPA typically develops between the ages of 40 and 80 years with mean age of onset younger than 65 years [21–23]. PPA has a devastating impact on the ability to communicate with others [24, 25], which makes the successful implementation of rigorous clinical trials that address speech and language symptoms a critical priority for optimizing quality of life for individuals with PPA and their families.

In PPA, there are a variety of aphasic deficits that can emerge at an individual level [15]. Three research-based symptom profiles, proposed by an international group of investigators, include the semantic variant (PPA-S) characterized by a decline in conceptual word knowledge; the agrammatic variant (PPA-G) characterized by a decline in the ability to accurately process and generate the underlying syntactic frame for language; and the logopenic variant (PPA-L) characterized by a decline in word finding and phonological abilities [26]. While distinct clinical presentations are observed in PPA, mixed or unclassifiable profiles also occur [27, 28]. Word retrieval, language processing, and language production difficulties are universal features of PPA regardless of subtype—although to varying levels of severity, and with differing sources of disruption [28]. For example, difficulties retrieving the correct word may result from conceptual impairments for some persons with PPA and from phonological sequencing impairments for others [29, 30]. Most persons with PPA have some level of difficulty understanding and generating connected speech (e.g., language beyond single words or sentences) even in the initial stages of the disease [28, 31]. Abilities decline over the course of the disease leaving language production fragmented and often reduced to a few frequently used words, formulaic utterances (e.g., “that’s all good”), vague terms (e.g., “thing”), and stereotypic words and phrases (e.g., “no-no-no”). As symptoms progress, the communication needs of persons with PPA may require the support of non-speech modes of communication (e.g., writing, pictures, texting, gestures) and in some cases computerized speech-generating devices [32–34]. In the face of these declines, communication partners carry the disproportionate burden for successful communication exchanges [35]. Because the inevitable progression of language impairment in PPA is complicated by declines in other cognitive and behavioral symptoms [22], interventions that address communication function and participation broadly are of particular importance.

Non-pharmacological interventions, delivered most often by a speech-language pathologist (SLP), are the primary intervention for speech, language, and communication impairments in PPA [36–38]. Because of its rarity, SLPs may have limited expertise with PPA [39]. While typically covered by insurance, SLP services are often terminated once a plateau in progress is reached leaving unmet needs as PPA symptoms progress. Alongside difficulties and delays in acquiring an accurate diagnosis, these factors limit access to PPA-specific assessments and interventions for speech, language, and communication challenges [40]. The last decade saw a growth in studies of speech-language interventions for persons with PPA [41–43]. However, a recent systematic review completed by the Progressive Disorders writing group of the Academy of Neurologic Communication Disorders and Sciences found that more than 90% of these studies implemented non-experimental, quasi-experimental designs, and single-case-study designs that lack robust randomization or control conditions [44]. Additionally, few studies followed participant outcomes beyond six months and thus the long-term effects of these interventions are unknown [44]. As a result, the overall evidence quality for speech-language interventions in PPA is still low, leaving patients and clinicians with limited clinical resources. Following our successful Stage I trial [42], we designed the CB2 clinical trial to fill gaps in the current clinical science, to expand the quality of evidence for clinical decision-making, and to advance speech-language interventions for persons with PPA. In the sections that follow, we provide a summary of the evidence that supports key components of the CB2 trial design.

#### Telehealth as a viable model for service delivery

Telehealth models for delivering speech-language interventions have demonstrated efficacy in several trials across clinical populations [45]. With few exceptions, this model has not been studied in persons with PPA [42, 46]. Telehealth models are particularly relevant for rare conditions, such as PPA, for which there is limited access to expert care [47]. Earlier studies showed that telehealth is a viable model for delivering speech-language therapies to individuals with PPA [42, 46]. In the current CB2 trial, our telehealth model delivers interventions synchronously through a video conference interface and is complemented by asynchronous home practice activities supported by the Communication Bridge™ web application. The web application provides access to speech and language practice exercises, educational resources, reminders/notes from the trial interventionist, and a motivational logging system for self-tracking completion of online home practice activities. In our pilot study, we showed that using a telehealth model to deliver synchronous and asynchronous speech-language intervention activities remotely is feasible [42].

#### Evidence in support of the Control arm intervention

In the control arm, participants receive an impairment-focused speech-language intervention [7]. Impairment-focused behavioral interventions for aphasia target underlying disrupted language and cognitive processes (e.g., lexical activation, implicit processing of phrasal movement in syntax structures) typically through structured, high repetition, intensive, therapeutic activities. These sometimes low-contextualized therapeutic activities are designed to elicit specific behaviors or linguistic targets that can be shaped toward a desired response through clinician feedback. Typical impairment-focused activities for language rehabilitation include naming picture targets facilitated by a cuing hierarchy, forming sentences that conform to a specific syntax target, or listening to sentences and responding to targeted content questions. Impairment-focused interventions propose to improve communication function by rehabilitating the underlying disrupted cognitive and/or language process and/or promoting optimal neural reorganization in the context of injury [8, 48]. Previous studies in PPA showed post-intervention improvements for impairment focused interventions targeting word retrieval [49]; agrammatism [50, 51]; alexia [52]; and connected speech/discourse [53]. The CB2 trial Control arm targets two language impairments common to persons with PPA using approaches that demonstrated efficacy in previous studies: (1) word retrieval using a semantic-phonological cueing hierarchy [54] and (2) language expression using Script Training [55] an intervention that capitalizes upon speech automaticity to generate larger units of scripted/scaffolded language exchanges in functional contexts [56].

#### Evidence in support of the Communication Bridge™—experimental arm intervention

Communication Bridge™ is a multi-component intervention that is consistent with the A-FROM framework [9, 57]. Briefly, A-FROM has four domains: language impairment(s); personal identity, attitudes, and feelings; communication environment; and participation [9, 57]. The FOURC model (Choose Communication Goal, Create Client Solution, Collaborate on a Plan, Complete and Continue) also aligns well with the Communication Bridge™ intervention framework first put forward by Rogalski et al. (2016), which incorporates collaborative goal setting, strategy development, environmental supports, and communication motivation/confidence development as therapy components [58]. Collectively, participation-focused intervention frameworks assume that to optimize a client’s ability to live with aphasia, interventions should address the four A-FROM domains in a multi-pronged approach that aligns with the communication goals of the client [7–9, 42, 58]. Participation-focused interventions target communication difficulties, construed broadly, within contextualized communication contexts (e.g., ordering food at the client’s favorite restaurant) [7]. In the CB2 experimental arm, barriers and limitations that prevent individuals from engaging in meaningful communication activities are addressed systematically through personalized communication strategies; communication partner training; modification of environmental factors that contribute to participation barriers (e.g., attitudes/knowledge of others in the communication environment); and targeted symptom-management education. While participation-focused interventions sometimes target retrieval of personally relevant word sets using drill-like activities, such approaches are more likely to include the use of adaptive communication aids/devices and collaborative problem solving of communication participation barriers. Like impairment-focused interventions, participation approaches are implemented systematically, and the clinician supplies performative feedback that shapes participant responses/actions toward a desired target/goal. However, in contrast to impairment-focused therapies, ‘success’ in participation-focused interventions is measured by the person’s ability to engage in everyday, personally meaningful, activities independent of changes in the nature or severity of the underlying disease or disrupted language/cognitive process [38]. Preliminary evidence in support of participation-oriented interventions for addressing PPA-related challenges comes from recent systematic reviews [44, 59] and from our Communication Bridge™ pilot study [42].

Collectively, the available literatures highlight the importance and feasibility of addressing communication participation limitations in persons with PPA through a joint synchronous and asynchronous telehealth model using evidence-informed interventions in both the control and experimental arms of the CB2 trial.

## Trial design and overview

### Objectives {7}

#### Aim 1

Determine the within-group response of the Experimental and Control interventions for individuals with PPA.

*Aim 1a*: To provide descriptive and quantitative characterizations of within-group responses to intervention. *Hypothesis 1*: The Experimental arm will show significant within-subject gains on standardized measures of communication participation, personalized GAS goals, and communication confidence while Control arm gains will be most robust in the language impairment performance measures

*Aim 1b*: Determine whether there are mediators that affect the magnitude of intervention response (e.g., home exercise compliance, PPA subtype, level of communication partner engagement). *Hypothesis 2*: We hypothesize participants’ home exercise compliance will be a significant mediating factor in intervention response, especially for the language performance measures.

#### Aim 2

Determine if a participation-focused, multi-component intervention approach (Experimental arm) for persons with PPA shows more favorable response in communication participation and communication confidence outcomes as compared to a dose-matched, impairment-focused intervention approach (Control arm) over a 12-month course of disease. *Hypothesis 3*: Participants in the Experimental arm will show more favorable communication outcomes relative to the Control arm.

### Trial design {8}

CB2 is a Stage 2 behavioral [1], randomized, parallel group, active-control, superiority trial designed to evaluate the efficacy of speech-language interventions for persons with PPA, specifically whether the Communication Bridge™ intervention (experimental arm) is superior to an impairment focused intervention in the Control arm. Participant dyads are allocated to the experimental arm and the control arm in a 3:2 ratio.

## Methods: participants, interventions, and outcomes

### Study setting {9}

All participants are enrolled through Northwestern University. Oregon Health & Science University is a participating site involved in data collection only. Northwestern University has primary administrative oversight for the study. Participants are recruited without any geographical restrictions. All trial visits are conducted synchronously using a video conference software platform.

### Eligibility criteria {10}

#### Participant dyads

Participant dyads are made up of an eligible participant with a PPA diagnosis and their communication partner. The trial requires the clinical diagnosis of PPA to be made by a neurologist and supported by the available medical records [22, 26, 60]. Participants must have an eligible communication partner who is willing to co-enroll into the trial. The CB2 trial defines a “communication partner” as an informal caregiver (typically a family member or friend) who has known the participant for more than 12 months, has close and regular contact with the participant, and provides emotional, communication, and/or activities of daily living support to the participant. By agreeing to enroll in the trial, communication partners consent to supporting the enactment of the core intervention components at the level required by the randomly assigned trial arm. Communication partners also serve as primary informants for measures of caregiver burden, communication participation, and communication difficulties. Inclusion and exclusion criteria are detailed in Table 1.

**Table 1:** Trial inclusion and exclusion criteria

**Applies to Both the Person with PPA and** **Co-Enrolled Healthy Adult Communication Partner**
Adults (18+ years of age). No maximum age limit.
English is primary language used in daily communication activities (self-report)
Adequate hearing (aided or unaided) for communicating with others in a crowded room (self-report)
Adequate vision (aided or unaided) for reading a newspaper, or other functional materials (self-report)
Able to pass technology screening^a^ and demonstrates sufficient knowledge for use of video conference and Communication Bridge^TM^ web application use (with or without training)
**Applies to Person with PPA Only**
Geriatric Depression Scale score ≤ 9
Mild-moderate language symptoms informed by a structured interview with a speech-language pathologist and by the following assessments: BDAE Spontaneous Speech task (‘Cookie Theft’ stimulus) scored using an adapted version of the WAB-R scoring system [61]; BDAE Repetition subtest: high probability words; NACC-FTLD Module noun naming subtest ≥ 80% (if score is 80 or lower, the NACC-FTLD word-picture matching is used to verify severity level).

*WAB-R* Western Aphasia Battery Revised [62], *BDAE *Boston Diagnostic Aphasia Exam [63], *NACC-FTLD* National Alzheimer’s Coordinating Center-Frontotemporal Lobar Degeneration assessment modules [64, 65]

^a^Denotes study-specific forms/instruments available with the CB2 Manual of Procedures [66]

#### Screening

Following medical record review and a brief screening battery (Table 1), participants complete a structured interview with a SLP who is not involved in delivering the intervention and who is blinded to trial arm allocation. The purpose of the SLP screening interview is to confirm the following: (1) that the prevailing barrier to communication participation is related to aphasia and thus targetable by the study interventions; (2) that the clinical impression of symptom severity is consistent with the screening assessment results [22]; and (3) that the dyad has the resources and suitable “readiness” for study participation. Readiness is defined by whether the dyad has the time, technology, emotional/family resources for an intensive 12-month study. A copy of the SLP screening interview form is available within the CB2 Manual of Procedures [67]. Screening and interview data are reviewed on a case-by-case basis by the administrative study team who make the final decision regarding trial eligibility. Implemented in our previous PPA observational studies, this multi-staged approach for verifying trial eligibility is critical because, in isolation, standardized assessment data may not accurately reflect the severity of aphasia, or other impairments, experienced by persons with PPA [68]. An overview of the screening and enrollment processes is provided in Table 2.

**Table 2:** Overview of study screening and enrollment processes

	Who	Procedures
**Pre-screening interview**	RA + PT/CP	Initial interview with PT/CP. Eligibility criteria reviewed. Verbal consent obtained for medical record review. RA (Research Assistant) works with PT/CP to request medical records.
**Medical record review**	AST	To confirm diagnosis and absence of other exclusionary medical indications.
**Technology check**	RA + PT/CP	PT/CP complete technology tasks with RA to verify sufficient technology skills and internet bandwidth for study sessions.
**Cognitive, sensory systems, and depression screening**	RA + PT/CP	PT/CP complete screening assessment tasks with RA to ensure participants meet eligibility criteria. Screening measures listed in Table 1.
**Secondary screening**	SLP	Non-treating SLP meets with dyad to confirm study readiness, PPA-related deficits /severity, and other relevant medical history information.
**Screening review and enrollment decision**	AST	The AST and other team members involved in screening process (RA and SLP) review all screening assessment results. AST makes the final decision to enroll through consensus.
**Consents/orientation**	RA + PT/CP	REDCap e-consent signature from PT+CP; Orientation to video conferencing platform and Communication Bridge web application. Complete demographic, health history, and questionnaires with PT. CP completes questionnaires independently and mails back to study team.

*RA* research assistant, *PT/CP* participant with PPA and their co-enrolled communication partner, *SLP* speech-language pathologist not involved in delivering interventions who is blinded to trial arm allocation, *AST* the administrative study team (authors EAS, ACR, and ER)

#### Trial interventionists

SLPs who have a clinical master’s degree in communication sciences and disorders and have earned a Certificate of Clinical Competence from the American Speech-Language Hearing Association administer the trial interventions. Trial interventionists have clinical experience in the assessment and treatment of persons with PPA, and experiences with speech-language interventions from both impairment and participation-focused theoretical frameworks.

### Who will take informed consent? {26a}

At the screening stage, participants provide verbal consent for medical record review. In cases where medical records are stored outside of the Northwestern Medicine system, participants request the release of their medical records and forward these to the study team. REDCap electronic consent [69] is used to obtain informed consent from each participant with PPA and their communication partner. An overview of the trial information and consent documentation is completed via video conference with research staff. As needed, study information is presented using simplified language and multi-modal communication supports to ensure sufficient information uptake for a valid informed consent. For persons with PPA, their care partner can support the consent process.

### Additional consent provisions for collection and use of participant data and biological specimens {26b}

Participants are asked to indicate their willingness to consent to three optional elements as part of their trial involvement: (1) consent to share audio and video data for scholarly publications, scientific presentations, and for teaching purposes; (2) consent to be re-contacted for future studies; and (3) consent to share de-identified data for use in other research studies conducted by faculty, staff, and students affiliated with the Northwestern University Mesulam Center for Cognitive Neurology and Alzheimer’s Disease. This trial does not involve collecting biological specimens for storage.

## Interventions

### Explanation for the choice of comparators {6b}

A parallel, active control, trial addresses concerns with delaying or denying the intervention to participants randomized to the control arm. This decision was motivated by several considerations. Importantly, the progressive nature of PPA creates ethical dilemmas related to withholding and/or delaying the intervention over a 12-month period or providing a less effective control intervention with limited or no efficacy. Additionally, recruitment and retention in dementia studies are improved by having an active control arm when compared to no-treat, delayed-treatment, sham, or education-only control conditions [70]. Further, we designed the control arm intervention such that it includes evidence-informed therapeutic components that can be dosed similarly to the experimental arm [70]. The control arm intervention was designed to intentionally contrast with the experimental arm on five key components:

1. *Theoretical perspectives underlying intervention approaches*. Whereas Communication Bridge™ is a multi-component intervention aligned with a participation-focused aphasia intervention framework, the control arm intervention is focused on improving underlying language impairments/processes and is aligned with an impairment-focused framework.
2. *The use of personalized training stimuli and education materials*. The experimental arm uses personalized training stimuli developed by the person with PPA and their communication partner with guidance from their interventionist. By contrast, the control arm uses the same pool of potential training stimuli across all participants. Thus, the two interventions differ along the dimension of personal salience. Similarly, participants in the control arm are exposed to general education on the nature, progression, and impairment profiles associated with PPA that is delivered in video format. In the experimental arm, participants view the same video but also receive personalized handouts/education tools from a library of core materials developed specifically for Communication Bridge™. The contrast in the inclusion of personally salient training stimuli and education materials extends to practice activities on the Communication Bridge™ web application.
3. *Dyad-centered design*. In the Communication Bridge™ intervention, both the person with PPA and their communication partner actively take part for the full duration of each synchronous intervention session, and activities are designed to optimize engagement by both participants. In contrast, communication partners in the control arm support the person with PPA but are only present during the first and final 5 min of each session as part of the weekly review and session wrap-up components. In the control arm, the communication partner is a supporting agent and not a direct recipient of the intervention. For both arms, participants complete home practice exercises on the Communication Bridge™ web application independently or with the support of their enrolled communication partner. In both trial arms communication partners may provide technology support to the person with PPA as needed.
4. *Personalized, goal-directed intervention sessions*. Participants in both arms develop personalized goals addressing participation in communication activities and communication participation limitations using a GAS approach [3, 4, 71–73]. In both trial arms, GAS goals are customized to each participant and developed through collaboration between participants and their interventionist. The person with PPA develops three GAS goals, while their communication partner develops one GAS goal. In the experimental arm, these goals are central to the intervention. Intervention activities, customized to each participant, are mapped onto specific GAS goals. By contrast, in the control arm, therapeutic activities stay fixed across participants and are not mapped onto individual GAS goals.
5. *Communication Bridge™ Web Application Content*. The Communication Bridge™ web application content differs between trial arms. Participants in both trial arms have unrestricted access to the application for home practice activities. In both arms, the number of visits and home exercise activity completions are tracked, with feedback provided to participants. However, in the experimental arm participants have an added “Reminders” feature where their interventionist can provide personalized weekly prompts around using communication strategies, reviewing assigned education handouts, and completing communication activities within their daily routines.

### Intervention description {11a}

The Communication Bridge™ Manual of Procedures is published on Northwestern DigitalHub [66] where details of the trial procedures and intervention protocols, by trial arm, can be found. A brief overview of the interventions provided to participants in each study arm follows.

#### Intervention service model

In both study arms, interventions are delivered through a combination of synchronous and asynchronous treatment activities. Synchronous sessions are delivered via a secure video-conferencing platform using a study-supplied laptop, a “plug-and-play” external microphone, and external speakers. Asynchronous treatment activities including home exercises and education activities are delivered via the Communication Bridge™ web application.

#### Intervention dosing

##### Synchronous therapy activity dosing

The dosing of each intervention is intended to be identical between trial arms and clinicians [66]. Participants in both arms complete a total of 15 synchronous, intervention sessions that are 55–75 min in length and are divided between two blocks with ten sessions in block 1 and five sessions in block 2. Intervention sessions are meant to occur once per week and are scheduled at a time of the participants’ choosing, typically on weekdays. Time spent in preliminary activities (e.g., weekly status check-ins, probe measures) versus targeted intervention activities is also intended to be equivalent between trial arms.

##### Asynchronous therapy activity dosing

Participants in both arms are encouraged to complete asynchronous home practice exercises on the Communication Bridge™ web application 30 min per day, 5 days per week. Web application logins and completed practice modules are tracked by the application. Participants and interventionists are provided feedback on the number of logins and completed practice activities upon logging into the Communication Bridge™ web application. The interventionist reviews these data with participants each week during the synchronous intervention session, alongside any challenges incurred during home practice. Participants have flexibility in when, and how, they complete the Communication Bridge™ web application practice activities.

#### Overview of core intervention approaches incorporated into the CB2 trial

A summary of the core interventions incorporated into each arm, and the features that distinguish their implementation approach is presented in the sections that follow. Table 3 provides a summary of the core intervention components by arm.

**Table 3:** Summary of intervention components by trial arm

Intervention component	Control arm	Experimental arm
Script training	Required	Optional
Word retrieval cuing hierarchy	Required	Optional
PPA “basics” educational video	Required	Required
Personalized communication strategy education and training	Not allowed	Required
Communication Bridge™ web application home practice activities	Required. Self-paced.	Required. Self-paced.
Personalized training stimuli for both synchronous and home practice activities.	Not allowed	Required
Therapeutic activities tailored to participant GAS goals	Not allowed	Required
Collaborative problem solving/counseling around barriers to communication participation	Not allowed	Required

Optional components are incorporated based on participant preference and whether the prescribed intervention approach is aligned with the participants’ GAS goals

##### Script training

Script training [55] is an intensive and structured intervention approach for persons with aphasia in which clients repeatedly practice phrases, sentences, and narratives formatted as monologues (e.g., describing the experience of living with PPA) or dialogues (e.g., exchange with a server in a restaurant). Script Training, which is based on the Instance Theory of Automatization [56], utilizes a variety of methods to support script learning including reading the script aloud repeatedly, producing the script following a clinician model, and generating the script spontaneously with varying levels of visual and/or auditory cues [74]. With intensive practice, persons with aphasia can retrieve portions of, or the entire script, fluently in the context of their daily activities [74, 75]. Studies of Script Training in aphasia secondary to stroke demonstrated generalization to spontaneous language beyond trained scripts [76].

CB2 trial guidelines for developing the training scripts and detailed procedures for implementing Script Training are in the Communication Bridge™ Manual of Procedures [66]. Regardless of the assigned trial arm, participants train on a base script set that includes one script describing what PPA is and what helps when communicating with a person who has PPA. From there, script training for participants in the control arm uses a fixed set of training stimuli (both monologues and dialogues) that are the same across participants. Scripts used in the control arm cover a range of topics important to persons with PPA that were informed by our pilot study. In contrast, training scripts used in the experimental arm are personalized to each participant and are linked to communication activities aligned with their GAS goals.

In both trial arms, the number of utterances in each script ranges from 8 to 12 utterances to minimize systematic differences in script difficulty across participants and arms. Scripts are trained consecutively. Participants are required to meet a threshold of 80% accuracy over two consecutive intervention sessions before adding a new script to the training set. In both trial arms, script training begins with the a base training script that is fixed across participants. An exception to this rule is applied in the experimental arm in which participants, when requested and when it aligns with their GAS goals, are allowed to introduce a new personalized script prior to mastering the base training script. Trained scripts are probed at each session. Scripts are scored by trained, independent outcome assessors using a multi-dimensional scoring system developed for the CB2 trial, that considers articulation, lexical retrieval, information content, grammar, and syntax structure accuracy [77]. To account for potential effects of script difficulty, each script is assigned a difficulty score using this multi-dimensional scoring system that can be used as a covariate in the statistical analysis. Script accuracy is scored as a percentage correct out of the maximum difficulty score assigned to the training script. Participants train on differing numbers of scripts depending on how quickly they reach proficiency for each script. In the control arm, participants are trained on up to five scripts. Scripts include two monologue and three dialogue scripts based on the principles described in Kaye and Cherney [77]. Participants in the experimental arm do not have a limit on the number of scripts that can be introduced into the formal Script Training activities. Additional “informal” scripts, not linked to specific GAS goals, may be developed as part of the experimental arm intervention but are practiced outside of a formal script training context and thus are not probed at each session.

##### Cuing hierarchy intervention for word retrieval

A 5-level, mixed phonological-semantic, word-retrieval cuing hierarchy intervention is implemented as an obligatory component in the control arm, but an optional component in the experimental arm depending on participant preference and their communication GAS goals. The cues in the hierarchy are organized from most to least supportive. In cuing hierarchy interventions, participants are repeatedly cued to generate a target word (or phrase) using a variety of cues (e.g., phonemic cues such as the first sound of a word, semantic cues such as a brief definition of a word) selected to stimulate activation and selection of lexical representations. The aim of such interventions is to strengthen the semantic, lexical, and/or phonological networks through repeated practice and targeted lexical retrieval. Cuing hierarchy interventions for word retrieval impairments are among the most frequently reported interventions for persons with PPA [41–43].

Regardless of trial arm, participants have a core set of training words that are developed for use in trial activities, including home practice activities on the Communication Bridge™ web application. In the control arm, these are the same words/phrases used in the cuing hierarchy. In the experimental arm, this core set can be trained using a cuing hierarchy intervention and/or can be incorporated into participation-focused activities. Regardless of arm or training approach, the core word list is probed at each training session using a naming to definition cue. Percent accuracy is recorded for each session and used to guide the addition of new words to the training set. The details of these procedures are provided in the Communication Bridge™ Manual of Procedures and are summarized below.

The potential set of training words/phrases for the control arm includes a list of 75 items taken from a previously published study for developing lexical training sets for persons with PPA and is augmented by 15 lower lexical frequency-of-occurrence words from the Multilingual Naming Test to achieve a potential training set of 90 words [78]. From these 90 words, an initial training set of 30 items is randomly selected from words that the participant was able to retrieve correctly in response to either a definition cue (max of 6 words) or first sound cue (max of 24 words). If the participant does not have enough stimulable words from which to draw for the training set, then the remaining words are selected randomly from the list of non-stimulable words. For the experimental arm, participants generate a list of 75 to 80 words that are salient to their everyday activities. Participants self-select the 30 words to use in the initial training set from two lists of words, one made up of words that they can retrieve correctly from definition (max of 6 words), and one made up of words that they can retrieve correctly with a phonemic cue (max of 24 words) and those for which they are not stimulable.

For participants in both arms, new words are introduced as proficiency is demonstrated on previous stimuli. Proficiency is defined as being able to retrieve the target word from a definition, in 5 s or less, over three consecutive sessions. Word accuracy is scored as a percentage correct for all words in the training set and is scored by trained, independent, outcome assessors. As the core training set is updated, the Communication Bridge™ web application is also updated so that the online, asynchronous word-retrieval practice activities match the stimuli incorporated into the synchronous therapy activities.

##### PPA “basics” education video

Participants in both arms view the same standardized PPA “basics” video with their interventionist and have the opportunity to ask questions related to the video content. This educational video provides an accessible overview of PPA including its pathophysiology, genetics, clinical symptom profiles, diagnosis, and progression. Participants view this video during the first intervention session and continue to have access to it over the duration of the trial.

##### Personalized strategy education and training

Only participants in the experimental arm receive communication strategy training and expanded disease/communication symptom education. The CB2 trial incorporates a variety of strategies based on the published literature across communication disorders [8, 37, 57, 79]. Common strategies taught in the experimental arm include the use of multi-modal communication supports such as teaching participants how to use layered and simultaneously deployed communication modes including gestures/body language, writing, texting, drawing, and speaking. Another aim of the Communication Bridge™ program is to teach participants how to build communication alliances with one another, and their family members/friends, to scaffold successful communication episodes whether at home or in the community. For example, clinicians may instruct the person with PPA to provide a non-stigmatizing cue to signal to their partner that they require increased support during a conversation with an unfamiliar group of people. The experimental arm also incorporates technology solutions to optimize communication. These can include the use of picture or story board web applications for conveying information or retelling events. The use of speech-to-text and text-to-speech native applications on smart devices can also be introduced to increase communication effectiveness for participants with better spelling/reading than speaking abilities. The library has 34 instructional documents and 39 tutorial videos covering a variety of communication strategies and technology solutions that are available to participants in the experimental arm. These are assigned by the interventionist based on the needs of participants and their goals. Additional personalized handouts are developed when needed to help achieve the participants' GAS goals. A comprehensive list of Communication Bridge™ library resources is published in the Manual of Procedures [66]. The strategies trained in each session, and the amount of time spent training each strategy, are documented using the study Run Sheet, provided in the Communication Bridge™ Manual of Procedures [66].

Communication strategies are taught through a variety of approaches including collaborative problem solving between the interventionist and participants, role-playing, strategy practice activities with performative feedback, and facilitated reflections, among other therapeutic approaches. Time spent in instruction for each strategy is documented by session to allow for monitoring the dosing of education and strategy training activities in relation to other core intervention activities. Education and strategy training activities are mapped to specific GAS goals allowing us to track which strategies were used to achieve specific goals.

##### Communication Bridge™ web application home practice activities

The Communication Bridge™ web application is an innovative part of the CB2 trial. It has four practice activities from which participants can select home exercises to complement their synchronous intervention sessions. Detailed in the Manual of Procedures, including screenshots of each exercise and the web application dashboard, these include Picture Cards a naming (word retrieval) exercise; Pronunciation Cards a word pronunciation exercise; the Word to Picture Matching exercise, and Script Practice exercise [66]. Regardless of the trial arm, the stimuli and targets populated to the exercise section of the web application match those used in the participant’s synchronous intervention sessions. One difference between the synchronous sessions and the web application is the use of picture stimuli in the Picture Cards exercise for word retrieval. For the control arm, picture stimuli are standardized across participants and items. In the experimental arm, participants provide personalized pictures representing targets in their core word training set, which can be augmented by the study team if needed. Thus, for the experimental arm, pictures used in the web application exercises are more representative of the participant's daily life. Handouts and video tutorial resources discussed earlier in the communication strategy and education core intervention description are also available through the web application. The availability of these materials is restricted for the control arm and unrestricted for the experimental arm. In the experimental arm, resources that are recommended by the interventionist, are posted weekly under each participant’s personalized “Reminders” page. The web application dashboard, visible to both groups, provides feedback regarding website visits and web exercise completion as well as upcoming study appointments [66]. In both trial arms, Communication Bridge™ web application activities are completed in a self-paced and self-directed manner [66].

### Criteria for discontinuing or modifying allocated interventions {11b}

There are no provisions for changing trial arm allocation or interventionists. However, participants may choose to withdraw at any time during the trial. Participants can be removed from the trial by the investigator if (1) their communication partner is no longer able to take part in the study; (2) they take part in other speech-language therapy services that interfere with the study interventions; (3) the study loses contact with the participant; (4) their participation in the trial would cause harm (e.g., extreme frustration with assessment or intervention tasks); or (5) the participant is no longer able to participate in required therapy sessions/tasks within the study visit scheduling rules. Examples of factors that could interfere with adhering to study scheduling rules include the onset of an acute or chronic interfering medical illness, or a change in living environment where access to the internet is no longer available. Participants who miss more than two visits will be withdrawn from the study at the end of the intervention block where the missed-visit threshold was crossed.

### Strategies to improve adherence to interventions {11c}

To optimize adherence to the interventions, all necessary study equipment is provided. Participants are provided with a technology study kit and a test materials kit, which are returned following the last study session. The technology kit includes a study-programmed laptop (Windows- or Mac-based depending on participant knowledge and/or preference), external USB microphone, external speakers, wireless mouse, ethernet cable for connecting the laptop directly to a modem, and a technology set-up guide to ensure consistency in hardware use and high-quality internet access across all participants. The test materials kit has manipulable objects needed for the Western Aphasia Battery-Revised (WAB-R) [62]. Other adherence strategies for minimizing intervention visit cancelations include flexible visit-scheduling windows that account for holiday time periods and other unexpected events, while still adhering to consistent dosing and assessment/intervention block timing. The detailed guidelines for visit scheduling, missed visits, and for rescheduling visits are in the Manual of Procedures [66].

Robust adherence to asynchronous practice exercise recommendations within the Communication Bridge™ web application is facilitated by automated feedback on the participant’s personalized “Achievements” dashboard (e.g., report of the number of times they visited the Communication Bridge™ web application, the number of exercises completed). Other intervention details (e.g., session times, home exercise reminders for the Experimental group) are posted to the participant’s personalized Communication Bridge™ web application to increase adherence and minimize data lost to missed visits. At each synchronous training session, the interventionist reviews the ‘Achievements’ page in the Communication Bridge™ application where the number of logins and exercise completions are tallied to assess compliance and the presence of any new, or recurring barriers, to practice (e.g., technology issues). The interventionist also reviews challenges with implementing the intervention at home, new concerns that affect communication goals, and potential scheduling issues. Additionally, at the beginning of each intervention block, the study team reviews the trial requirements with participants and assesses for the presence of any intervening factors such as changes in health status or participation in other activities that could compromise the CB2 trial.

To reduce technology barriers that could affect asynchronous practice compliance, the study team provides technology tutorials to participants at the beginning of the trial and are available to provide technology support to both participants and interventionists as needed throughout the duration of their study participation. Written technology handouts are provided to participants to aid in completing weekly video sessions and using the Communication Bridge™ web application.

### Relevant concomitant care permitted or prohibited during the trial {11d}

Participants with PPA are asked to withhold from co-enrolling in a study or clinical intervention that could interfere with the CB2 trial results. These include participation in other behavioral or neuromodulation interventions for language, cognition, or speech symptoms. Otherwise, care continues as normal. If the need for non-competing, services arise (e.g., audiology, vision assessment, dysphagia assessment), the administrative study team and interventionist provide support to coordinate those services with the trial intervention. Participation in caregiver education and support groups is allowed provided these do not have a primary speech-language therapy focus (e.g., communication strategy training).

### Provisions for post-trial care {30}

There are no formal post-care provisions. All participants receive a general resource handout following discharge. Participants continue to have access to the Communication Bridge™ web application for self-directed practice and education materials following study completion. However, web application materials are not modified or updated after completing the trial.

### Outcomes {12}

In keeping with the A-FROM framework, the primary study outcomes are related to communication confidence and participation and include (1) a standardized measure of communication confidence, the Communication Confidence Rating Scale for Aphasia (CCRSA) [5]; (2) a standardized measure of communication participation in everyday activities, the Communication participation Item Bank (CPIB) [2]; and (3) performance on person-centered communication participation goals generated at baseline through goal attainment scaling procedures [3, 4]. Other language impairment outcomes, detailed under the core interventions section, include percent accuracy on trained words and scripts (probed each session). The primary study outcomes of communication confidence and participation are aligned with the theoretical model underpinning Communication Bridge™. The A-FROM [9, 57] and WHO-ICF [7] models identify communication confidence and participation as distinct therapy targets for minimizing disability and optimizing quality of life for persons with aphasia. Previous studies in communication disorders highlight the importance of intentionally targeting the construct of communication participation because changes in underlying impairments may not translate to improvements in functional status or engagement in everyday activities [2, 80]. All outcomes reported below are measured at five timepoints during the trial: baseline; post intervention block 1 (PIE 1); at 6 months post enrollment, prior to beginning intervention block 2; post intervention block 2 (PIE 2), and at the end of the study (~ 12 months post enrollment).

#### Communication confidence

Communication confidence is measured using a single outcome. The CCRSA measure is a Likert scale, that asks persons to rate how confident they feel communicating in different situations both in the home (e.g., understanding a television program) and in the community [5, 6, 81]. The CCRSA theoretically ranges from 0 to 100 with higher ratings indicating more confidence. The psychometric properties of the measure, originally validated in persons with aphasia secondary to stroke, have been published previously [5]. Currently, there are no published psychometric properties for the CCRSA in persons with PPA. The CCRSA was used as the primary outcome in our pilot study [42]. Participants complete a paper version of the CCRSA scale in self-paced format, then meet with a research coordinator to review and verify responses. Paper copies of the response form are returned to the study team via mail. At each assessment point (following baseline), participants are provided with response forms that have their most recent responses marked in red ink. Providing participants with their most recent responses minimizes recall bias by providing an anchor to support a more accurate self-assessment of current status relative to their previous communication confidence levels.

#### Communication participation

Communication participation outcomes include both the CPIB and person-centered GAS goals.

##### Communication Participation Item Bank (CPIB)

The CPIB measure is a 10-item, Likert scale that asks persons to rate how their condition interferes with participating in daily activities (e.g., talking to people you know/do not know, securing a turn in a fast-moving conversation). The psychometric properties of the measure, developed for persons with communication disorders broadly, have been published previously [2]. Both raw and T-scores are available. The CPIB T-score has a theoretical mean of 50 and standard deviation of 10 with higher ratings reflecting higher levels of participation. Like the CCRSA, participants complete the CPIB in self-paced, paper format and then meet with a research coordinator to review their responses. As with the CCRSA, participants are provided with their responses from their most recent previous assessment to anchor their ratings. For CCRSA and CPIB, the mean of these outcomes at each visit, as well as the mean change from baseline to each of the follow-up visits will be compared within and between arms.

##### Goal Attainment Scaling (GAS) goals

Participant-centered goals are developed during the baseline evaluation sessions. The GAS development procedure for the CB2 trial is detailed in the Communication Bridge™ Manual of Procedures [66]. Briefly, GAS goals are developed collaboratively in a process that involves the assigned interventionist, the person with PPA, and their communication partner. GAS goals are developed by triangulating information gathered from a dynamic assessment clinical interview conducted by the interventionist [82]; the Assessment of Living with Aphasia (ALA) [83] a standardized assessment based on the A-FROM model used to identify individualized barriers to communication participation in persons with aphasia; and the Social Networks Inventory (SNI) [84] a standardized inventory for characterizing the size and scope of a person’s communication circle(s) and their communication modes (i.e., the methods and modalities they use to communicate with others such as gestures, words, and texting).

All GAS goals are centered on communication-related activities addressable by speech-language therapies. A total of four GAS goals are developed for each participant dyad, three that target communication from the person with PPA’s perspective and one that targets communication from the communication partner’s perspective. Each GAS goal is written such that the current level of function/performance is anchored at ‘0’. At its extremes, the GAS scale is anchored at + 2 (improvement from baseline) and − 2 (decline from baseline). Levels + 1 and + 2 reflect incremental improvements with + 2 reflecting the participant’s self-stated “best” anticipated outcome. Levels − 1 and − 2 reflect incremental levels of decline with − 2 reflecting the participant’s “worst” anticipated outcome. Written descriptions are developed by the interventionist for each anchor that describes the functional/performance expectations at each level, which are verified with both members of the participant dyad prior to finalizing. In cases where participants exceed performance anchors at + 2 or − 2, they are assigned a score of + 3 or − 3 based on the direction of change, improvement or decline, respectively. At each assessment timepoint, GAS goals are evaluated by a non-treating SLP assessor who is familiar with the dyad but is blinded to trial arm allocation. At each timepoint, the SLP assessor conducts a dynamic assessment clinical interview with both participants and uses this information to score the current functional/performance status for each GAS goal based on the descriptions provided for each anchor.

To determine trial outcomes, participants will be classified into performance bins (decrease in scores, no change in scores, or increase in scores) based on within person change at each of the following time points: (1) initial intervention effect - baseline to post-intervention evaluation following therapy block one (PIE1); (2) maintenance of initial intervention effect - PIE1 to 12 months post enrollment; and (3) net effect of intervention over the full trial duration - baseline to 12 months post enrollment. For CCRSA and CPIB, binning will be based on clinically meaningful change thresholds as defined in the relevant literature. For GAS, binning will be as improvement or increase, no change, and decline or decrease based on direction of GAS goal score changes. Trial “success” will be defined as a statistically significant difference (odds ratios favoring experimental arm), comparing the combined percentage change from participants binned to the “no change” and “increased” categories between arms, for any single primary outcome measure, at any one of the critical timepoints. Effectively, we will determine trial success by comparing percentage change for those participants who remain stable, or who demonstrate improvements, at each time point on the CCRSA, CPIB, and GAS goals. Defining “success” as stable or improved performance is important for behavioral interventions in neurodegenerative disorders where the intrinsic state of the condition is one in which progressive, incremental, decline is expected. Thus, maintaining stability of baseline functional status (i.e., slowing symptom decline) in the face of neurodegeneration is an important therapeutic target [85].

### Participant timeline {13}

We enrolled our first participant on May 3, 2018. We expect to enroll the final participant in the first quarter of 2022, with all study visits completed by March of 2023. Time from first study contact to enrollment is variable and depends on several factors. Depending on the time required to retrieve medical records, time from screening to enrollment can be as short as 10 days (about 1 and a half weeks). However, in some cases when added testing is needed to confirm a diagnosis of PPA or to rule out other confounding conditions, the time to enrollment may be longer (Table 2).

The total study time commitment for participants is approximately 12 months. Participants complete assessment visits prior to therapy blocks one and two, at baseline and 6 months, respectively. Post-intervention evaluations (PIE) are conducted following each intervention block. A final evaluation and a post-study debriefing interview are conducted following the final intervention session visit at month 12 following enrollment. A listing of the assessment measures/tasks completed at each evaluation block is in the Communication Bridge™ Manual of Procedures [66]. In the Appendix is a SPIRIT guideline figure with details of the study timeline and core protocol activities.

### Sample size {14}

The enrollment target is 90 PPA dyads that include a person with mild to moderate PPA and their co-enrolled communication partner, with 54 dyads allocated to the experimental arm and 36 dyads to the control arm. Using their baseline assessment data, medical records, and neuropsychological baseline data participants with PPA are assigned to one of three research subtypes semantic, agrammatic, and logopenic using previously published consensus criteria [26]. Allowing for an estimated attrition rate of 6% (i.e., loss of five participants), the planned minimum sample size is 85 participants (51 experimental arm and 34 control arm). These sample sizes have 80% power to detect a between arm mean difference of 0.70 standard deviations (SDs) assuming a two-tailed Type I error rate of 2.5%. There is 80% power to detect a within arm mean change of 0.40 SDs in the Experimental arm and 0.50 SDs in the control arm assuming a two-tailed Type I error rate of 5%. Pilot data from our Stage I trial [42] indicated a within arm CCRSA effect size of 0.52 SDs for the change from baseline to post speech-language intervention sessions at two months post-baseline so that the trial was powered to detect expected within arm changes in CCRSA.

### Recruitment {15}

Participants are recruited through several mechanisms, without geographical restriction, including the research registry at the Mesulam Center for Cognitive Neurology and Alzheimer’s Disease at Northwestern University, clinician referral (e.g., physician, speech-language pathologist, social worker), and self-referral. Study awareness efforts include direct mailing (e-mail and physical mail to targeted audiences), website listings, professional and patient-focused listservs, non-governmental organizations (e.g., Alzheimer’s Association, Association for Frontotemporal Degeneration), clinical trial finder websites, word of mouth, networking and presentations within the National Institute on Aging (NIA)–funded Alzheimer’s Disease Research Centers, clinical registration through Clinicaltrials.gov, and promoting the study at professional and lay-community conference/educational events. Recruitment status relative to planned targets is reviewed monthly by the administrative study team and PI for adjusting recruitment plans as needed. A recruitment committee tracks recruitment efforts, and their referral success rates.

## Assignment of interventions: allocation

### Sequence generation {16a}

The randomization schedule was generated using SAS software (Version 9.4 SAS System for Windows, Copyright (c) 2002–2012 by SAS Institute Inc., Cary, NC, USA). A permuted block randomization schedule was stratified by trial interventionist and subtype with 15 individuals from each subtype (semantic, agrammatic, logopenic) randomized to the experimental (*n* = 9) or control (n = 6) arm for each of two interventionists. Within each interventionist and subtype, the 15 randomized individuals consisted of three blocks of five participants, three allocated to the experimental arm and two to the control arm.

### Concealment mechanism {16b}

The project manager and statistician have primary access to the randomization schedule. While the remainder of the study team is blinded to the allocation schedule, the full schedule for the study was formatted in a single spreadsheet and revealed to the project manager at the study outset.

### Implementation {16c}

A study team member who is not involved in the intervention delivery completes the enrollment process with participants. The project manager assigns participants to interventions following enrollment and unblinds the intervention arm to the interventionist following the baseline assessment.

## Assignment of interventions: blinding

### Who will be blinded {17a}

Trial arm concealment is preserved by using unique participant identifiers that do not contain any participant or trial arm label information. Regardless of the study arm, outcome assessors use identical forms and procedures for collecting and recording outcome measure data, which minimizes risk of revealing intervention assignment during scoring. However, while these forms are labeled by study identifier only, assessors involved in scoring the CCRSA and CPIB measures may not be strictly blinded to participant arm beyond the baseline assessment. They may be able to re-identify participants based on familiarity developed through study and technology support activities. Importantly, the outcome assessor involved in the GAS goal scoring is blinded at all timepoints. Central study lists linking participant number and intervention assignment are stored on REDCap [69] and maintained exclusively by the project manager. This list is not shared with the outcome assessors or statistical team to facilitate intervention group concealment for participant-level data. When participant-specific discussions regarding intervention or clinical care are required, meetings are held separately with each interventionist to minimize sources of bias that could affect intervention implementation between clinicians or across arms. Participants remain blinded to trial arm allocation for the duration of the study protocol and following discharge. Interventionists are blinded to group allocation for the baseline assessment and the creation of GAS goals but are unblinded to group assignment before the first intervention session. Because the trial interventionists administer interventions in both study arms, they are unable to remain blinded to study arm beyond the baseline assessment. They remain blinded to the primary outcomes data at all time points. Interventionists are not involved in the scoring of primary study outcomes.

### Procedure for unblinding if needed {17b}

The statistician, analysts, PI, and core administrative team members will be unblinded for the purposes of completing interim analyses of the primary and other outcome data for the Data Safety and Monitoring Board. The unblinding of participants whose data are included in the interim analysis will be completed in REDCap by the project manager. Other circumstances that may necessitate unblinding include significant adverse events that have potential to be related to the study protocol, participant withdrawal, or other study-safety-related issues.

## Data collection and management

### Plans for assessment and collection of outcomes {18a}

Assessors receive extensive training in administering and scoring study assessments and measures. A clinical neuropsychologist (author SW) assesses competency in administering most of the neuropsychological assessments. Competency in the administration of the remaining patient report and speech-pathology scales and measures, along with establishing and writing GAS goals, is evaluated by the project manager and/or author ACR (a trained and registered SLP). The assessments and measures used to collect participant descriptive data are detailed in the Communication Bridge™ Manual of Procedures [66]. With few exceptions, all study assessments and measures are administered and scored in accordance with their published manuals and/or instructions. Deviations from published protocols, and/or bespoke administration or scoring rules specific to the CB2 trial, are detailed in the Communication Bridge™ Manual of Procedures [66] and in recent publications [61].

### Plans to promote participant retention and complete follow-up {18b}

The CB2 trial employs several strategies that are consistent with best practices for improving participant retention in dementia clinical trials [70, 86, 87]. These include the use of flexible schedules that allow week-to-week changes in study visit appointments to accommodate participant work and personal schedules. Technology barriers to retention are reduced by supplying all required study devices and implementing technology that is portable. This allows participants to travel and change locations without missing study sessions. Robust, remote, technology support provided by the study team and through Northwestern University Information Technology further reduces knowledge barriers that could negatively impact retention. Access to the Communication Bridge™ web application and communication practice activities between intervention blocks provides motivation for study participants and keeps them connected with the study protocol between synchronous intervention sessions, further reducing study attrition. Because there are no costs to participants, there are few financial barriers to study participation. When a participant withdraws from the study, all primary and other outcomes collected prior to leaving the study remain in the dataset and, if willing/able, participants may be asked to complete the final debriefing interview.

### Data management {19}

Data management procedures are overseen by the project manager and compliance is reviewed regularly during scheduled administrative study team meetings. Study data are managed using REDCap electronic data capture tools hosted at Northwestern University [69]. REDCap (Research Electronic Data Capture) is a secure, web-based software platform designed to support data capture for research studies, providing (1) an intuitive interface for validated data capture; (2) audit trails for tracking data manipulation and export procedures; (3) automated export procedures for seamless data downloads to common statistical packages; and (4) procedures for data integration and interoperability with external sources. Data management procedures are outlined in the study protocol [66] and are summarized here. Prior to data entry, all paper-based forms are checked for missing and/or ambiguous responses (e.g., two responses circled for a single item). Missing or ambiguous responses trigger a call to the participant to determine if the response was omitted intentionally or in error. If produced in error the intended response is clarified. All measures, except for patient-reported outcomes, are scored twice by independent assessors. Automated checks in the database flag out-of-range values and missing data for required fields. Study sessions are audio/video recorded via the video conference software for later response verification, missing data recovery, and fidelity assessments.

### Confidentiality {27}

Personal information about potential and enrolled participants is exchanged through secure scan/fax (via Box Capture), phone call, secure REDCap link, and/or secure email exchange. When required by non-local interventionists, de-identified paper copies of study records are couriered (via FedEx or other secure shipping services) to the study coordinating site (Northwestern University) with package tracking. Email exchanges with participants are conducted via university-secured e-mail servers. Identifiable data, and study documents that have identifiable information, are stored on secure Northwestern University network servers that are backed up hourly. Recruitment mailing lists are stored separately from trial data and are kept by a central committee within the Mesulam Center that oversees recruitment for all center research activities. Contact information (physical addresses and phone numbers) needed to ship equipment to participants are stored securely in REDCap, on Northwestern University network servers, and when in physical form in locked filing cabinets located in research-dedicated spaces. Upon study completion, computer devices are mailed back to the study and are cleaned to remove any file traces or identifiable information before deploying to a new participant. Participants and interventionists log into remote study sessions using a unique video conference link that reduces opportunities for logging into another participant’s session. Security settings are optimized for the video conference software to prevent breaches from non-study participants. As an added layer of security, participants and interventionists are provided with an ethernet cable and are required to plug directly into their modem for all treatment sessions both for optimal connectivity, and to minimize potential hardware device hacking via non-secure WIFI connection. Alternatively, interventionists may connect to sessions from a secure university network connection. Each participant has a personal login for the Communication Bridge™ web application that provides access only to their study resources. To further preserve confidentiality, interventionists have a personal login that allows them access to their participant data only.

### Plans for collection, laboratory evaluation, and storage of biological specimens for genetic or molecular analysis in this trial/future use {33}

This is not applicable as there will be no biological specimens collected.

## Statistical methods

### Statistical methods for primary and other outcomes {20a}

An intent-to-treat data analysis is planned. The primary study outcomes are the Communication Confidence Rating Scale for Aphasia (CCRSA), the Communication Participation Item Bank (CPIB), and Goal Attainment Scaling (GAS). Intervention effect will be analyzed using a marginal linear model of the follow-up measure (SAS PROC MIXED) for means of CCRSA and CPIB or PROC GENMOD for odds ratios of GAS and for binned outcomes (decrease, no change or increase) with the randomized group as the primary independent variable and the baseline value of that measure as a covariate [88]. This analysis of covariance model will account for repeated measures and heterogeneity of variance and adjusts for the baseline value of the outcome. Subtype will also be included as a covariate. This marginal linear model has randomized group, subtype, time, and the group by time interaction as fixed effects. The significance of the adjusted group difference at the follow-up time of interest will be determined using *p* < 0.05 for each outcome. Additional group comparisons will be done for the absolute and percent change from baseline to each follow-up time. A standardized area under the curve will quantify the average percent change from baseline over the entire follow-up period to better examine patterns of change over time between the two groups. Within group analyses will compare the mean change or percent change using a one-sample *t* test from the marginal model. Within-group effect size will be calculated as the mean change divided by the standard deviation of the change.

### Interim analyses {21b}

One interim analysis is planned, once half of the participants (*n* = 45) have completed their 12-month follow-up visits. O’Brien-Fleming boundaries in an alpha spending function will be used for the interim analysis [89]. An overall two-sided alpha level of 0.05 is assumed for each outcome. For all measures, the interim and final decision rules would be to declare a difference in the interim analysis if *p* < 0.0058 and in the final analysis if *p* < 0.0442. The administrative study team, the statistician, the Data Safety and Monitoring Board (DSMB), and the NIA Program Officer will have access to the interim analysis results. After reviewing the results, the DSMB will recommend continuing or terminating the study to the NIA Program Officer (February 2022). There are no pre-determined “stop” rules for the study.

### Methods for additional analyses (e.g., subgroup analyses) {20b}

Baseline comparisons between arms will be done for standardized evaluation measures specified in the inclusion criteria. For outcomes, measures of outcome success will be based on thresholds of change in outcome over the follow-up period and will be determined separately for each outcome. Secondary analyses will determine baseline factors that are related to the success of each outcome. Further analyses will compare change over time among trial outcomes, as well as other longitudinal cognitive and language measures. Effect sizes for change over time will be the metric used to compare different cognitive and language measures. These analyses will determine the rate at which different cognitive and language dimensions are changing. In mediation analyses, potential mediating measures that will be examined in relation to different outcomes include level of depression, home exercise compliance, and communication partner engagement. These analyses will identify intermediate factors affected by the trial intervention that drive intervention-based changes in outcomes.

### Methods in analysis to handle protocol non-adherence and any statistical methods to handle missing data {20c}

Although dropout at the 12-month time point is expected to be under 10%, the primary statistical analysis will also be conducted using inverse probability of censoring weighting to account for bias that may be incurred by missing data due to dropout [90].

### Plans to give access to the full protocol, participant level-data and statistical code {31c}

The intervention protocol, anonymized summary data, and any statistics software scripts/routines used to analyze data will be available to the scientific community at large in conjunction with publication of the final trial results. The trial protocol will be made available through the Communication Bridge™ collection on Northwestern University’s DigitalHub. De-identified participant-level data and associated metadata will be made available to the scientific community, following an approved study collaboration request.

### Composition of the coordinating center and trial steering committee {5d}

Northwestern University is the coordinating center. The coordinating center oversees the fiscal management of the award, ethics approvals, subcontract awards, and other financial and oversight responsibilities. The data management team is comprised of the core administrative study team including authors ER, EAS, and ACR and statistician AWR. A Data Safety and Monitoring Board (DSMB), described in {21a} monitors study safety, protocol compliance, and achievement of study aims and endpoints. A fidelity monitoring team led by author ACR oversees treatment implementation and procedural fidelity.

### Composition of the data monitoring committee, its role and reporting structure {21a}

The DSMB is comprised of a statistician with clinical trials expertise, a neuropsychologist with dementia clinical trials expertise, and a communication disorders scientist with expertise in participation-focused outcome measures and communication-focused interventions. The composition of the DSMB was approved by the study funder (National Institutes on Aging, NIA) and is responsible for monitoring clinical trial compliance and data safety. The DSMB receives a written report and meets with the administrative study team including authors ER, EAS, ACR, and statistician AWR twice annually to review trial conduct and progress, fidelity data, a summary reporting of adverse events, and summary reporting of any interim trial issues or concerns. Between these meetings, the DSMB Chair and the Program Officer receive adverse event reporting per NIA guidelines and are consulted on other trial conduct and major trial modifications as required. The administrative study team and statistician report to the DSMB (led by the Chair), who reports to the NIA Program Officer overseeing the grant. The approved DSMB charter is available through Northwestern DigitalHub [91].

### Adverse event reporting and harms {22}

Adverse events are defined and reported following the *NIA Adverse Events and Serious Adverse Events Guidelines* [92]. All events are recorded by the study team member first notified of the event using a study-specific reporting form. Within 24–48 h (depending on NIA policy), adverse events are reviewed by the administrative study team and the study team member reporting the event. Other ad hoc members are consulted as appropriate (i.e., social worker, study neurologist, statistician). For each adverse event, an action plan is developed and monitored by the administrative study team. The tracking of adverse events and follow-up actions are overseen by the project manager and the PI. Adverse event reports are entered into REDCap and paper copies are stored in a study-specific Adverse Event binder, securely locked in a filing cabinet in project manager office.

### Frequency and plans for auditing trial conduct {23}

The CB2 trial employs a robust intervention fidelity plan that is modeled on standards reported for web-based behavioral trials by Eaton and colleagues (2011) [93]. Interventionists and study personnel complete a detailed protocol training prior to collecting data or implementing interventions. Knowledge of the study protocol is evaluated through demonstrating competency during simulated study activities (e.g., mock GAS goal development, completing study documentation from training recordings). Interventionists must demonstrate essential competencies in study documentation, participant assessment, GAS goal development, and intervention deployment (e.g., mock assessments, therapy sessions, and goal setting) before seeing participants. Fidelity is assessed in an ongoing manner, as data are collected.

For each interventionist, 100% of study sessions for their first five participants, in each study arm (10 participants/clinician), are subjected to offline fidelity assessment (using audio and video recordings of sessions). An additional randomly selected sample, made up of 25% of study sessions in each study arm, is subjected to offline fidelity assessments. Additional “spot-checks” are performed as study documentation is entered, and as fidelity questions arise in the course of deploying the study. Fidelity assessors are either non-treating SLPs or trained research assistants. Assessors evaluate procedural fidelity (i.e., evaluating whether each step of the implementation protocol is implemented correctly), documentation fidelity (i.e., whether case report forms are complete and correct), and theoretical fidelity (i.e., whether the session implementation and interventionists’ feedback align as intended with the assigned trial arm).

Standardized checklists and criteria are used by assessors for each fidelity domain. Procedural and documentation fidelity items are scored using a binary rating of “1” (completed correctly) or “0” (incomplete or not completed correctly) and are reported as the proportion of required items performed correctly. Theoretical fidelity is scored using a Likert scale centered on “0” where aspects of the protocol (e.g., interventionists feedback, how responses were shaped) are scored such that scores on the positive side of the scale indicate procedures and feedback that are focused on function and participation in life activities and scores on the negative side of the scale indicate procedures and feedback that are more directed toward modifying underlying skilled behaviors or impairments, and ‘0’ scores indicating an even balance of impairment-focus and participation-focus approaches in the session. Theoretical fidelity is reported as a single summed score across all the assessment items with the expectation that experimental arm sessions will be polarized toward positive scores (e.g., more participation-focused) and that control arm sessions will be polarized toward negative scores (e.g., more impairment-focused). Our two-pronged approach minimizes and monitors for potential contamination between the experimental and control arms [93]. This is critical given the risk for bias created by the pragmatic need to have interventionists delivering treatments in both trial arms.

The overall fidelity plan also involves regular team meetings on topics such as general PPA education and study-specific topics including strategies/approaches for addressing specific language/communication difficulties, strategies for developing GAS goals with participants, strategies for intervention session management, documentation, among other ad hoc topics. Study protocol “tips” are distributed to the team via email weekly and highlight key protocol elements to ensure ongoing compliance to intervention implementation procedures. Session documentation tracking forms, that include session start and end times, are entered weekly and used to monitor dosing compliance across participants and arms. Session duration compliance reports are distributed to the administrative study team and interventionists weekly. These reports flag the aggregate number of in- and out-of-compliance sessions, by interventionist, and are used to follow up weekly on potential threats to dosing compliance within and between trial arms, and interventionists. Two or more out-of-compliance sessions within any reporting period, or two consecutive weeks with an out-of-compliance session, will trigger a meeting with the interventionist and the administrative study team to discuss the source of the dosing violations and strategies for meeting the targeted dosing levels. Regular fidelity compliance meetings with the full study team (following the DSMB meeting schedule) are conducted in which fidelity assessment data are reviewed with trial interventionists. As part of these meetings, and also ad hoc when the need arises, action plans to address fidelity concerns are developed and implemented. These plans include, but not exclusively, group retraining, targeted individualized re-training with a trial interventionist, or increased monitoring efforts. In addition to a comprehensive fidelity plan, our use of a detailed study procedures manual [66], and our ongoing and regular diligence to fidelity monitoring, supports the overall rigor of the CB2 trial and allows the study team to address potential concerns early.

### Plans for communicating important protocol amendments to relevant parties (e.g., trial participants, ethical committees) {25}

Unless required to address a participant emergency or time-critical issue, all changes to the protocol will be reviewed and approved by the Northwestern University Institutional Review Board (IRB) prior to their implementation. Significant changes to the protocol, required for day-to-day study operations, must be approved by the administrative study team and statistician AWR. Major changes to the protocol design ) requires approval of the DSMB prior to implementing. Unless a modification to the protocol is required in response to increased participant risk, major protocol modifications are reported to the DSMB at regularly scheduled meetings. If the protocol change is in response to increased participant risk, or represents a substantial change in the study conduct, the DSMB Chair and the funding Program Officer are contacted by the PI within the timelines set forth by the funding body. The clinicaltrials.gov protocol registration will be updated by the project manager within 14 days (about 2 weeks) of major protocol changes.

### Dissemination plans {31a}

Investigators will report results in summary form, at the end of the trial, on ClinicalTrials.gov. Study results will be communicated primarily through scientific presentations, lay-audience conferences, patient education websites, public media, and peer-reviewed publications. Publication of peer-reviewed manuscripts will be delayed until the trial is completed. However, studies addressing non-trial direct research questions using the baseline trial data may be published prior to the study conclusion.

## Discussion

PPA is a rare clinical neurodegenerative dementia syndrome. Although awareness and research studies focused on PPA have increased, the evidence-base for non-pharmacologic treatment remains limited. Single case studies are the predominant design in published clinical trials of speech-language interventions, in part because of enrollment challenges given limited access to rigorously diagnosed cohorts of persons with PPA and geographical restrictions imposed by in-person trial designs. These single-case designs offer important and detailed insights into the types of interventions and strategies that may be useful in PPA, [41–43]. However, these study designs are unable to provide unequivocal evidence of efficacy which is necessary for effectiveness trials and implementation. Our telehealth service delivery model provides an essential vehicle to advance PPA behavioral interventions. The experimental arm utilizes a multi-component, dyadic intervention in which both the person with PPA and their co-enrolled communication partner are active intervention recipients. The active control arm was modeled off the psycholinguistic frameworks more commonly seen in single-case study designs in the existing literature. Namely, it is a non-dyadic intervention in which the person with PPA is the active intervention recipient and their communication partner is in a supporting role with therapies addressing word retrieval and language production. The primary goal of the clinical trial is to determine if the experimental arm is superior to the control arm. However, a secondary and important by-product of the CB2 trial will be data from a robustly characterized cohort of persons with PPA, collected under rigorous conditions, that can be used to evaluate the efficacy of speech-language interventions delivered in both trial arms for persons with PPA. The impact of these data should not be overlooked as they will yield important insights examining why interventions work, which strategies are optimal, and for whom. Collectively, the information gained from the trial will fill critical gaps necessary to advance effectiveness trials for speech-language interventions in PPA.

Although the telehealth model facilitates the ability to conduct the intervention, recruitment and enrollment remain cumbersome. Clinician referrals have been the most efficient and successful source of participants, highlighting the importance of teamwork in conducting clinical trials. We utilize several recruitment strategies to raise awareness about the study including digital marketing, direct outreach to relevant clinicians, and presentations to targeted audiences. Our most common barriers in screening are incomplete or insufficient medical reports. The COVID-19 pandemic did not require pausing enrollment but slowed enrollment pace since non-urgent medical care was limited thereby reducing referrals from specialty clinics.

The study is not without limitations and potential biases. To keep up with the enrollment pace, our trial requires the use of more than one treating interventionist, and both are required to provide treatment in the control and experimental arms. This is a potential source of bias if a clinician favors one treatment over the other. We mitigate this bias by providing a detailed manual of procedures [66], regular team meetings, weekly protocol reminder emails, and thorough fidelity assessments that closely monitor both procedural and theoretical fidelity.

One of the primary tenets of the experimental arm is the tailored nature of the intervention, which is focused on addressing life participation goals of the participants. To meet and assess this aim we used GAS as a primary outcome, which requires setting goals for each participant in both arms. As described in the protocol, these goals are at the center of the Experimental intervention but do not guide intervention activities in the control arm. GAS goal literature suggests that the practice of setting goals is therapeutic in and of itself and could potentially lead to unanticipated therapeutic benefit in the control arm that will need to be considered in the interpretation of the trial results [94]. The COVID-19 pandemic brought unexpected challenges in that it restricted opportunities for social and community engagement, which may disproportionally impact the experimental arm given its focus on communication confidence and participation in everyday activities.

While these considerations are important to consider, we have designed the trial in a way to maximize rigor and reproducibility in hopes of advancing behavioral interventions for persons with PPA.

## Trial status

The CB2 trial is currently operating under version 12.0 of the protocol (March 27, 2020). The first enrollment was May 3, 2018. Recruitment will be completed by December 2021. The final enrollment is scheduled for February 2022 with all participants completing the study by March 2023.

## Figures and Tables

**Fig. 1:**
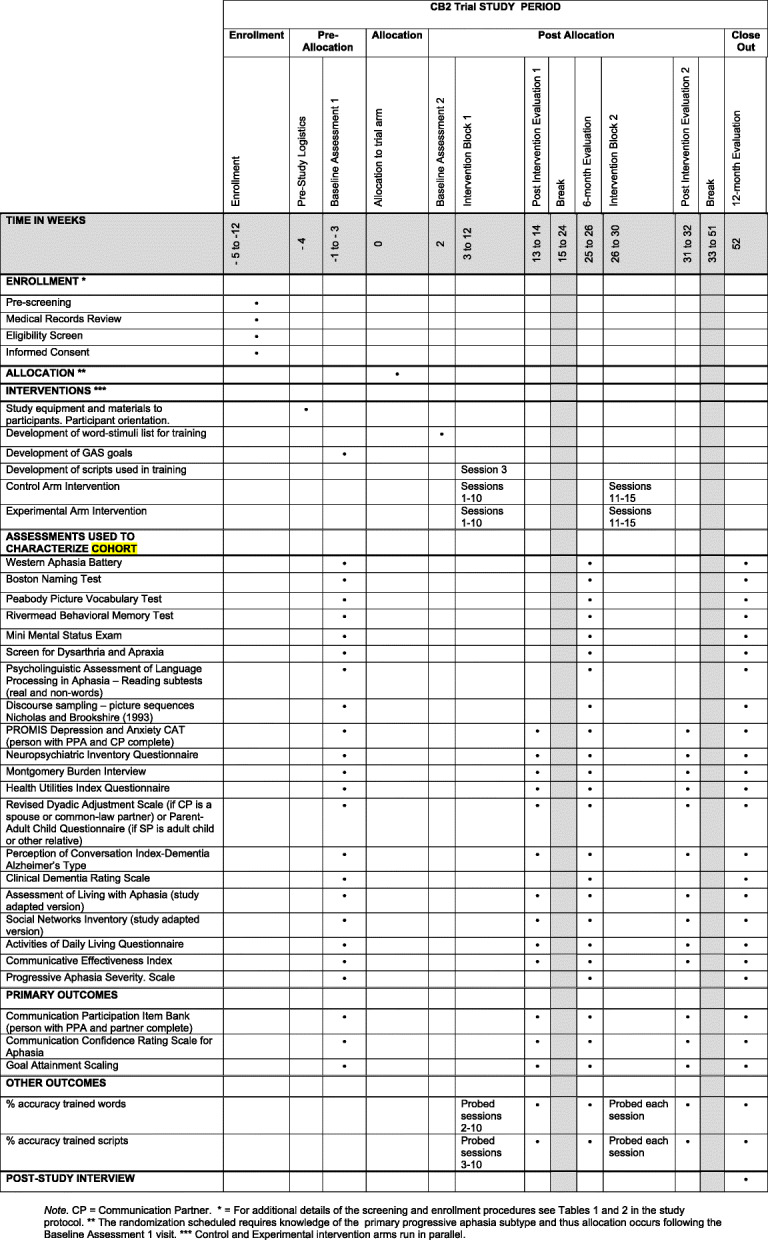
SPIRIT figure of study timeline. Note: CP, Communication Partner. * For additional details of the screening and enrollment procedures see Tables 1 and 2 in the study protocol. ** The randomization scheduled requires knowledge of the primary progressive aphasia subtype and thus allocation occurs following the baseline assessment 1 visit. *** Control and experimental intervention arms run in parallel.

## References

[1] Onken LS, Carroll KM, Shoham V, Cuthbert BN, Riddle M (2014). Reenvisioning Clinical Science. Clin Psychol Sci.

[2] Baylor C, Yorkston K, Eadie T, Kim J, Chung H, Amtmann D (2013). The Communicative Participation Item Bank (CPIB): item bank calibration and development of a disorder-generic short form. J Speech Lang Hear Res.

[3] Ottenbacher KJ, Cusick A (1990). Goal Attainment Scaling as a method of clinical service evaluation. Am J Occup Ther.

[4] Schlosser RW (2004). Goal attainment scaling as a clinical measurement technique in communication disorders: a critical review. J Commun Disord.

[5] Babbitt EM, Heinemann AW, Semik P, Cherney LR (2011). Psychometric properties of the Communication Confidence Rating Scale for Aphasia (CCRSA): Phase 2. Aphasiology..

[6] Cherney LR, Babbitt EM, Semik P, Heinemann AW (2011). Psychometric Properties of the Communication Confidence Rating Scale for Aphasia (CCRSA): Phase 1. Top Stroke Rehabil.

[7] Threats TT (2008). Use of the ICF for clinical practice in speech-language pathology. Int J Speech Lang Pathol.

[8] Kagan A, Simmons-Mackie N (2007). Beginning with the end: outcome-driven assessment and intervention with life participation in mind. Top Lang Disord.

[9] Kagan A, Simmons-Mackie N, Rowland A, Huijbregts M, Shumway E, McEwen S, Threats T, Sharp S (2008). Counting what counts: A framework for capturing real-life outcomes of aphasia intervention. Aphasiology..

[10] Simmons-Mackie N, Damico JS (2001). Intervention outcomes: a clinical application of qualitative methods. Top Lang Disord.

[11] Khayum B, Rogalski E (2018). A life participation approach to primary progressive aphasia intervention. Semin Speech Lang.

[12] Morhardt D, Weintraub S, Khayum B, Robinson J, Medina J, O’Hara M, Mesulam M, Rogalski EJ (2015). The CARE Pathway Model for Dementia. Psychiatr Clin North Am.

[13] Howick J, Chalmers I, Glasziou P, Greenhalgh T, Heneghan C, Liberati A, Moschetti I, Phillips B, Thronton OG, & Hodgkinson M. “The Oxford Levels of Evidence 2” https://www.cebm.ox.ac.uk/resources/levels-of-evidence/ocebm-levels-of-evidence.

[14] Mesulam MM (2001). Primary progressive aphasia. Ann Neurol.

[15] Rogalski EJ, Mesulam MM (2009). Clinical trajectories and biological features of primary progressive aphasia (PPA). Curr Alzheimer Res.

[16] Mesulam MM, Coventry C, Kuang A, Bigio EH, Mao Q, Flanagan ME, Gefen T, Sridhar J, Geula C, Zhang H, Weintraub S, Rogalski EJ (2021). Memory Resilience in Alzheimer Disease With Primary Progressive Aphasia. Neurology.

[17] Rogalski E, Cobia D, Harrison TM, Wieneke C, Weintraub S, Mesulam MM (2011). Progression of language decline and cortical atrophy in subtypes of primary progressive aphasia. Neurology.

[18] Weintraub S (1990). Primary Progressive Aphasia. Arch Neurol.

[19] Mesulam MM, Coventry C, Bigio EH, Geula C, Thompson C, Bonakdarpour B (2021). Nosology of Primary Progressive Aphasia and the Neuropathology of Language. Adv Exp Med Biol.

[20] Spinelli EG, Mandelli ML, Miller ZA, Santos-Santos MA, Wilson SM, Agosta F, Grinberg LT, Huang EJ, Trojanowski JQ, Meyer M, Henry ML, Comi G, Rabinovici G, Rosen HJ, Filippi M, Miller BL, Seeley WW, Gorno-Tempini ML (2017). Typical and atypical pathology in primary progressive aphasia variants. Ann Neurol.

[21] Mesulam M, Wicklund A, Johnson N, Rogalski E, Léger GC, Rademaker A, Weintraub S, Bigio EH (2008). Alzheimer and frontotemporal pathology in subsets of primary progressive aphasia. Ann Neurol.

[22] Mesulam MM, Rogalski EJ, Wieneke C, Hurley RS, Geula C, Bigio EH, Thompson CK, Weintraub S (2014). Primary progressive aphasia and the evolving neurology of the language network. Nat Rev Neurol.

[23] Mesulam MM, Weintraub S, Rogalski EJ, Wieneke C, Geula C, Bigio EH (2014). Asymmetry and heterogeneity of Alzheimer’s and frontotemporal pathology in primary progressive aphasia. Brain.

[24] Coyle-Gilchrist ITS, Dick KM, Patterson K, Vázquez Rodríquez P, Wehmann E, Wilcox A, Lansdall CJ, Dawson KE, Wiggins J, Mead S, Brayne C, Rowe JB (2016). Prevalence, characteristics, and survival of frontotemporal lobar degeneration syndromes. Neurology..

[25] Grossman M (2010). Primary progressive aphasia: clinicopathological correlations. Nat Rev Neurol.

[26] Gorno-Tempini ML, Hillis AE, Weintraub S, Kertesz A, Mendez M, Cappa SF, Ogar JM, Rohrer JD, Black S, Boeve BF, Manes F, Dronkers NF, Vandenberghe R, Rascovsky K, Patterson K, Miller BL, Knopman DS, Hodges JR, Mesulam MM, Grossman M (2011). Classification of primary progressive aphasia and its variants. Neurology.

[27] Mesulam M, Wieneke C, Rogalski E, Cobia D, Thompson C, Weintraub S. Quantitative Template for Subtyping Primary Progressive Aphasia. Arch Neurol. 2009;66(12). 10.1001/archneurol.2009.288 PMC2796598 20008661

[28] Mesulam MM, Wieneke C, Thompson C, Rogalski E, Weintraub S (2012). Quantitative classification of primary progressive aphasia at early and mild impairment stages. Brain.

[29] Henry ML, Wilson SM, Babiak MC, Mandelli ML, Beeson PM, Miller ZA, Gorno-Tempini ML (2016). Phonological Processing in Primary Progressive Aphasia. J Cogn Neurosci.

[30] Mack JE, Chandler SD, Meltzer-Asscher A, Rogalski E, Weintraub S, Mesulam MM, Thompson CK (2015). What do pauses in narrative production reveal about the nature of word retrieval deficits in PPA?. Neuropsychologia..

[31] Wilson SM, Henry ML, Besbris M, Ogar JM, Dronkers NF, Jarrold W, Miller BL, Gorno-Tempini ML (2010). Connected speech production in three variants of primary progressive aphasia. Brain..

[32] Mooney A, Bedrick S, Noethe G, Spaulding S, Fried-Oken M (2018). Mobile technology to support lexical retrieval during activity retell in primary progressive aphasia. Aphasiology..

[33] Weintraub S, Rogalski E, Shaw E, Sawlani S, Rademaker A, Wieneke C, Mesulam MM (2013). Verbal and nonverbal memory in primary progressive aphasia: The Three Words-Three Shapes Test. Behav Neurol.

[34] Fried-Oken M (2008). Augmentative and Alternative Communication Treatment for Persons With Primary Progressive Aphasia. Perspect Augment Altern Commun.

[35] Davies K, Howe T (2020). Experiences of Living With Primary Progressive Aphasia: A Scoping Review of Qualitative Studies. Am J Alzheimers Dis Other Dement.

[36] Carthery-Goulart MT, Silveira ADCD, Machado TH, Mansur LL, Parente MADMP, Senaha MLH (2013). Nonpharmacological interventions for cognitive impairments following primary progressive aphasia: A systematic review of the literature. Dement Neuropsychologia.

[37] Khayum B, Wieneke C, Rogalski E, Robinson J, O’Hara M (2012). Thinking Outside the Stroke: Treating Primary Progressive Aphasia (PPA). Perspect Gerontol.

[38] Kortte KB, Rogalski EJ (2013). Behavioural interventions for enhancing life participation in behavioural variant frontotemporal dementia and primary progressive aphasia. Int Rev Psychiatry.

[39] Taylor C, Kingma RM, Croot K, Nickels L (2009). Speech pathology services for primary progressive aphasia: Exploring an emerging area of practice. Aphasiology.

[40] Kim E, Figeys M, Hubbard H, Wilson C (2018). The Impact of Aphasia Camp Participation on Quality of Life: A Primary Progressive Aphasia Perspective. Semin Speech Lang.

[41] Farrajota L, Maruta C, Maroco J, Martins IP, Guerreiro M, De Mendonça A (2012). Speech Therapy in Primary Progressive Aphasia: A Pilot Study. Dement Geriatr Cogn Disord Extra.

[42] Rogalski EJ, Saxon M, McKenna H, Wieneke C, Rademaker A, Corden ME, Borio K, Mesulam MM, Khayum B (2016). Communication Bridge: A pilot feasibility study of Internet-based speech-language therapy for individuals with progressive aphasia. Alzheimers Dement.

[43] Volkmer A, Rogalski E, Henry M, Taylor-Rubin C, Ruggero L, Khayum R, Kindell J, Gorno-Tempini ML, Warren JD, Rohrer JD (2020). Speech and language therapy approaches to managing primary progressive aphasia. Pract Neurol.

[44] Wauters LD, Ballard KJ, Clark HM, Croot K, Dial HR, Duffy JR, Grasso SM, Kim E, Kohley L, Milman LL, Rogalski EJ, Schaffer KM, Henry ML (2021). Behavioral Treatment for Primary Progressive Aphasia & Primary Progressive Apraxia of Speech: A Systematic Review Clinical Aphasiology.

[45] Weidner K, Lowman J (2020). Telepractice for Adult Speech-Language Pathology Services: A Systematic Review. Perspect ASHA Special Interest Groups.

[46] Dial H, Hinshelwood H, Grasso S, Hubbard HI, Gorno-Tempini M-L, Henry M (2019). Investigating the utility of teletherapy in individuals with primary progressive aphasia. Clin Interv Aging.

[47] Groft SC, Posada De La Paz M. Preparing for the Future of Rare Diseases: Springer International Publishing; 2017. p. 641–8. 10.1007/978-3-319-67144-4_34. 10.1007/978-3-319-67144-4_34 29214596

[48] Simmons-Mackie N, Kagan A (2007). Application of the ICF in Aphasia. Semin Speech Lang.

[49] Croot K, Taylor C, Abel S, Jones K, Krein L, Hameister I, Ruggero L, Nickels L (2015). Measuring gains in connected speech following treatment for word retrieval: a study with two participants with primary progressive aphasia. Aphasiology..

[50] Machado TH, Campanha AC, Caramelli P, Carthery-Goulart MT (2014). Brief intervention for agrammatism in Primary Progressive Nonfluent Aphasia: A case report. Dement Neuropsychol.

[51] Thompson CK, Barbieri E, Mack JE, Wilkins A, Xie KY (2021). Plasticity of sentence processing networks: evidence from a patient with agrammatic variant of primary progressive aphasia (PPA). Neurocase.

[52] Getz H, Snider S, Brennan D, Friedman R (2016). Successful remote delivery of a treatment for phonological alexia via telerehab. Neuropsychol Rehabil.

[53] Whitworth A, Cartwright J, Beales A, Leitão S, Panegyres PK, Kane R (2018). Taking words to a new level: a preliminary investigation of discourse intervention in primary progressive aphasia. Aphasiology..

[54] Henry ML, Hubbard HI, Grasso SM, Dial HR, Beeson PM, Miller BL, Gorno-Tempini ML (2019). Treatment for Word Retrieval in Semantic and Logopenic Variants of Primary Progressive Aphasia: Immediate and Long-Term Outcomes. J Speech Lang Hear Res.

[55] Cherney LR, Halper AS, Holland AL, Cole R (2008). Computerized script training for aphasia: preliminary results. Am J Speech Lang Pathol.

[56] Logan GD (1988). Toward an instance theory of automization. Am Psychol Assoc.

[57] Kagan A (1998). Supported conversation for adults with aphasia: methods and resources for training conversation partners. Aphasiology.

[58] Haley KL, Cunningham KT, Barry J, de Riesthal M (2019). Collaborative Goals for Communicative Life Participation in Aphasia: The FOURC Model. Am J Speech Lang Pathol.

[59] Volkmer A, Spector A, Meitanis V, Warren JD, Beeke S (2020). Effects of functional communication interventions for people with primary progressive aphasia and their caregivers: a systematic review. Aging Ment Health.

[60] Mesulam MM (2003). Primary Progressive Aphasia — A Language-Based Dementia. N Engl J Med.

[61] Rao LA, Roberts AC, Schafer R, Rademaker A, Blaze E, Esparza M, Salley E, Coventry C, Weintraub S, Mesulam MM, Rogalski E. The Reliability of Telepractice Administration of the Western Aphasia Battery-Revised in Persons With Primary Progressive Aphasia. Am J Speech Lang Pathol. 2022;31(2):881-95. 10.1044/2021_AJSLP-21-00150. 10.1044/2021_AJSLP-21-00150 PMC9150668 35175852

[62] Kertesz A (2007). Western Aphasia Battery-Revised Pearson.

[63] Kaplan E, Goodglass H, Weintraub S (1983). Boston Naming Test.

[64] Staffaroni AM, Weintraub S, Rascovsky K, Rankin KP, Taylor J, Fields JA, et al. Uniform data set language measures for bvFTD and PPA diagnosis and monitoring. Alzheimers Dement. 2021;13(1). 10.1002/dad2.12148 PMC7896637 33665340

[65] Gefen T, Teylan MA, Besser L, Pollner E, Moshkovich A, Weintraub S (2020). Measurement and characterization of distinctive clinical phenotypes using the Frontotemporal Lobar Degeneration Module (FTLD-MOD). Alzheimers Dement.

[66] Rogalski E, Roberts A, Communication Bridge Authors. CB FINAL HANDBOOK 11_14_19.pdf DigitalHub.: Galter Health Sciences Library & Learning Center; [Available from: https://digitalhub.northwestern.edu/collections/ce29f2be-9a64-4717-973b-ff531c2a93ee.

[67] Rogalski E, Roberts A, Communication Bridge Authors Screen Template.dox Digital Hub: Galter Health Sciences Library & Learning Center [Available from: https://digitalhub.northwestern.edu/collections/ce29f2be-9a64-4717-973b-ff531c2a93ee.

[68] Weintraub S, Rogalski E, Mesulam M (2016). The Behavioral Neurology of Dementia.

[69] Harris PA, Taylor R, Thielke R, Payne J, Gonzalez N, Conde JG (2009). Research electronic data capture (REDCap)—A metadata-driven methodology and workflow process for providing translational research informatics support. J Biomed Inform.

[70] Elliott CL (2020). Together We Make the Difference: National Strategy for Recruitment and Participation in Alzheimer’s and Related Dementias Clinical Research. Ethn Dis.

[71] Jennings LA, Ramirez KD, Hays RD, Wenger NS, Reuben DB (2018). Personalized Goal Attainment in Dementia Care: Measuring What Persons with Dementia and Their Caregivers Want. J Am Geriatr Soc.

[72] Chew J, Chong M-S, Fong Y-L, Tay L (2015). Outcomes of a multimodal cognitive and physical rehabilitation program for persons with mild dementia and their caregivers: a goal-oriented approach. Clin Interv Aging.

[73] Sohlberg MM, Mateer CA (2001). Cognitive rehabilitation: An integrative neuropsychological approach.

[74] Kaye RC, Cherney LR (2016). Script Templates. Top Lang Disord.

[75] Youmans G, Youmans SR, Hancock AB (2011). Script Training Treatment for Adults With Apraxia of Speech. Am J Speech Lang Pathol.

[76] Cherney LR, Kaye RC, Lee JB, Sv V (2015). Impact of Personal Relevance on Acquisition and Generalization of Script Training for Aphasia: A Preliminary Analysis. Am J Speech Lang Pathol.

[77] Roberts A, Esparza M, Aveni A, Rogers L, Rao L, Mooney A, Khayum R, Blaze E, Rogalski E (2020). A novel multidomain script training scoring approach for individuals with primary progressive aphasia. American Speech-Language and Hearing Association Conference Virtual.

[78] Ivanova I, Salmon DP, Gollan TH (2013). The Multilingual Naming Test in Alzheimer's Disease: Clues to the Origin of Naming Impairments. J Int Neuropsychol Soc.

[79] Savundranayagam MY, Moore-Nielsen K (2015). Language-based communication strategies that support person-centered communication with persons with dementia. Int Psychogeriatr.

[80] Baylor C, Oelke M, Bamer A, Hunsaker E, Off C, Wallace SE, Pennington S, Kendall D, Yorkston K (2017). Validating the Communicative Participation Item Bank (CPIB) for use with people with aphasia: an analysis of differential item function (DIF). Aphasiology..

[81] Babbitt EM, Cherney LR (2010). Communication confidence in persons with aphasia. Top Stroke Rehabil.

[82] Power E, Thomas E, Worrall L, Rose M, Togher L, Nickels L, Hersh D, Godecke E, O'Halloran R, Lamont S, O'Connor C, Clarke K (2015). Development and validation of Australian aphasia rehabilitation best practice statements using the RAND/UCLA appropriateness method. BMJ Open.

[83] Simmons-Mackie N, Kagan A, Victor JC, Carling-Rowland A, Mok A, Hoch JS, Huijbregts M, Streiner DL (2014). The assessment for living with aphasia: Reliability and construct validity. Int J Speech Lang Pathol.

[84] Treadwell TW, Leach E, Stein S (1993). The social networks inventory: A diagnostic instrument measuring interpersonal relationships. Small Group Res.

[85] Epperly T, Dunay MA, Boice JL (2017). Alzheimer Disease: Pharmacologic and Nonpharmacologic Therapies for Cognitive and Functional Symptoms. Am Fam Physician.

[86] Together We Make the Difference (2018). National Strategy for Recruitment and Participation in Alzheimer’s and Related Dementias Clinical Research.

[87] Local Diverse Working Group (National Institute on Aging). Alzheimer's disease and related dementias clinical studies recruitment planning guide. 2019. National Institute on Aging. https://www.nia.nih.gov/sites/default/files/2019-05/ADEAR-recruitment-guide-508.pdf.

[88] Frison L, Pocock SJ (1992). Repeated measures in clinical trials: Analysis using mean summary statistics and its implications for design. Stat Med.

[89] Demets DL, Lan KKG (1994). Interim analysis: The alpha spending function approach. Stat Med.

[90] Seaman SR, White IR (2013). Review of inverse probability weighting for dealing with missing data. Stat Methods Med Res.

[91] Rogalski E, Roberts A, Communication Bridge Authors. DSMP_v9.pdf DigitalHub.: Galter Health Sciences Library & Learning Center.

[92] NIA Adverse Event and Serious Adverse Event Guidelines. 2018. https://www.nia.nih.gov/sites/default/files/2018-09/nia-ae-and-sae-guidelines-.pdf.

[93] Eaton LH, Doorenbos AZ, Schmitz KL, Carpenter KM, McGregor BA (2011). Establishing Treatment Fidelity in a Web-Based Behavioral Intervention Study. Nurs Res.

[94] Fishman KN, Ashbaugh AR, Swartz RH (2021). Goal Setting Improves Cognitive Performance in a Randomized Trial of Chronic Stroke Survivors. Stroke..

